# Involving society in science: Reflections on meaningful and impactful stakeholder engagement in fundamental research

**DOI:** 10.15252/embr.202154000

**Published:** 2021-11-04

**Authors:** Helen Garrison, Marta Agostinho, Laura Alvarez, Sofie Bekaert, Luiza Bengtsson, Elisabetta Broglio, Digna Couso, Raquel Araújo Gomes, Zoe Ingram, Emma Martinez, Ana Lúcia Mena, Dörthe Nickel, Michael Norman, Inês Pinheiro, Marta Solís‐Mateos, Michela G Bertero

**Affiliations:** ^1^ Vetenskap & Allmänhet Stockholm Sweden; ^2^ EU‐LIFE Alliance of Research Institutes Advocating for Excellent Research in Europe Barcelona Spain; ^3^ CeMM Research Center for Molecular Medicine of the Austrian Academy of Sciences Vienna Austria; ^4^ VIB,Ghent University Ghent Belgium; ^5^ Max Delbrück Center for Molecular Medicine in the Helmholtz Association (MDC) Berlin Germany; ^6^ Centre for Genomic Regulation (CRG) Barcelona Institute of Science and Technology Barcelona Spain; ^7^ Universitat Autònoma de Barcelona Barcelona Spain; ^8^ Instituto Gulbenkian de Ciência (IGC) Oeiras Portugal; ^9^ Babraham Institute Cambridge UK; ^10^ Institut Curie PSL Research University Paris France; ^11^ Institut Curie PSL Research University Nuclear Dynamics Unit Paris France; ^12^ University Pompeu Fabra (UPF) Barcelona Spain

**Keywords:** Economics, Law & Politics, History & Philosophy of Science

## Abstract

Open Science calls for transparent science and involvement of various stakeholders. Here are examples of and advice for meaningful stakeholder engagement.

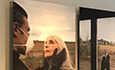

The concepts of Open Science and Responsible Research and Innovation call for a more transparent and collaborative science, and more participation of citizens. The way to achieve this is through cooperation with different actors or “stakeholders”: individuals or organizations who can contribute to, or benefit from research, regardless of whether they are researchers themselves or not. Examples include funding agencies, citizens associations, patients, and policy makers (https://aquas.gencat.cat/web/.content/minisite/aquas/publicacions/2018/how_measure_engagement_research_saris1_aquas2018.pdf). Such cooperation is even more relevant in the current, challenging times—even apart from a global pandemic—when pseudo‐science, fake news, nihilist attitudes, and ideologies too often threaten social and technological progress enabled by science. Stakeholder engagement in research can inform and empower citizens, help render research more socially acceptable, and enable policies grounded on evidence‐based knowledge. Beyond, stakeholder engagement is also beneficial to researchers and to research itself. In a recent survey, the majority of scientists reported benefits from public engagement (Burns *et al*, 2021). This can include increased mutual trust and mutual learning, improved social relevance of research, and improved adoption of results and knowledge (Cottrell *et al*, 2014). Finally, stakeholder engagement is often regarded as an important factor to sustain public investment in the life sciences (Burns *et al*, 2021).

Stakeholder engagement in research can inform and empower citizens, help render research more socially acceptable and enable policies grounded on evidence‐based knowledge

Here, we discuss different levels of stakeholder engagement by way of example, presenting various activities organized by European research institutions. Based on these experiences, we propose ten reflection points that we believe should be considered by the institutions, the scientists, and the funding agencies to achieve meaningful and impactful stakeholder engagement.

## How can stakeholder engagement be achieved?

Importantly, the recent COVID‐19 crisis has emphasized the need for improving public understanding of the scientific process. The time needed between fundamental discoveries and application, and the evolution of scientific knowledge through questioning, revisiting and self‐correcting acquired knowledge, are concepts that need to be broadly communicated. Greater public understanding of the scientific process will not only contribute to openness and increased trust in science, but also help to develop critical, analytical, and transparent attitudes.

Advocates for public engagement have been arguing for decades about the need to overcome a deficit view, according to which the public lacks sufficient information about science and technology. Instead, to increase scientific literacy, encourage participation, and foster public acceptance of science, engagement should be a dialogue in which different stakeholders actively participate (Stilgoe *et al*, 2014). Another development is the move away from trying to achieve consensus to eliciting and accepting diverse views (Mohr, 2008). Stakeholder engagement can thus help to acquire scientific knowledge, create attitudes that value science as part of cultural development, and enable an active role of citizens and scientists in social debates (Godin & Gingras, 2000).

Advocates for public engagement have been arguing for decades about the need to overcome a deficit view, according to which the public lacks sufficient information about science and technology.

There are multiple frameworks to define stakeholder engagement in research. To better characterize the examples, we adopted Gabriele Bammer's model, which defines different levels of engagement with different stakeholders as well as the responsibilities of researchers at each level (https://i2insights.org/2020/01/07/research‐modified‐iap2‐spectrum/). It encompasses five stages from informing—one‐way communication—to collaborating and empowering via two‐way or even “multi‐way” dialogue (Table 1). It should be stressed that different levels often overlap and complement each other and that all are of value depending on the specific objectives and audiences.

**Table 1:** The table illustrates the different levels of stakeholder engagement, from informing to empowering, as well as researchers' responsibilities at each level.

	Inform	Consult	Involve	Collaborate	Empower
Stakeholder participation goal	Researchers provide stakeholders with balanced and objective information to assist them in understanding the research	Researchers obtain stakeholder feedback on the research	Researchers work directly with stakeholders to ensure that stakeholders concerns and aspirations are consistently understood and considered in the research	Researchers partner with stakeholders for salient aspects of the research	Researchers assist stakeholders in conducting their own research
Promise made to stakeholders from researchers	We will keep you informed	We will keep you informed, listen to and acknowledge your concerns and aspirations and provide feedback on how your input influenced the research	We will work with you to ensure your concerns and aspirations are directly reflected in the research, and we will provide feedback on how your input influenced the research	We will look to you for advice and innovation in designing and conducting the research and incorporate your advice and recommendations to the maximum extent possible	We will provide advice and assistance as requested in line with your decisions for designing and conducting your research, as well as for implementing the findings

## Examples of stakeholder engagement across different levels

Here, we present examples of stakeholder engagement in the life sciences at different levels and on different scales, from informing to collaborating and empowering. Stakeholders can represent diverse groups of people, from patients, artists, pupils at school, policy makers, or other researchers to citizens in general. The selected examples demonstrate how this layering of engagement approaches can be used to engage citizens of any background. Through identification and discussion of the benefits and challenges, we propose ten reflection points to achieve meaningful and impactful stakeholder engagement.

The examples—activities carried out by several European research institutes that participate in the EU‐LIFE alliance and different Europe‐wide projects, such as LifeTime Initiative and ORION Open Science—highlight the main outcomes, the challenges, the main contributions by researchers, and stakeholders as well as any potential tensions. They were chosen based on their originality, their success, and how well they represent the different levels of engagement. Although the selected framework helps to categorize different activities, we recognize that engagement represents a continuum, from unidirectional communication to two‐way dialogue, and many of the activities fall within multiple categories and represent multiple levels of engagement. In fact, the portfolio of science communication activities provided by the institutions involved in writing this paper was much larger. For example, we specifically omitted important, but commonly undertaken school outreach activities.

## Inform

Even if informing—whereby researchers disseminate knowledge about discoveries and technologies in an unbiased way—represents the first step of engagement, it is still a necessary activity. Informing often presents fewer barriers to non‐expert audiences and acts as an entry point to more engagement.

**Figure 1:**
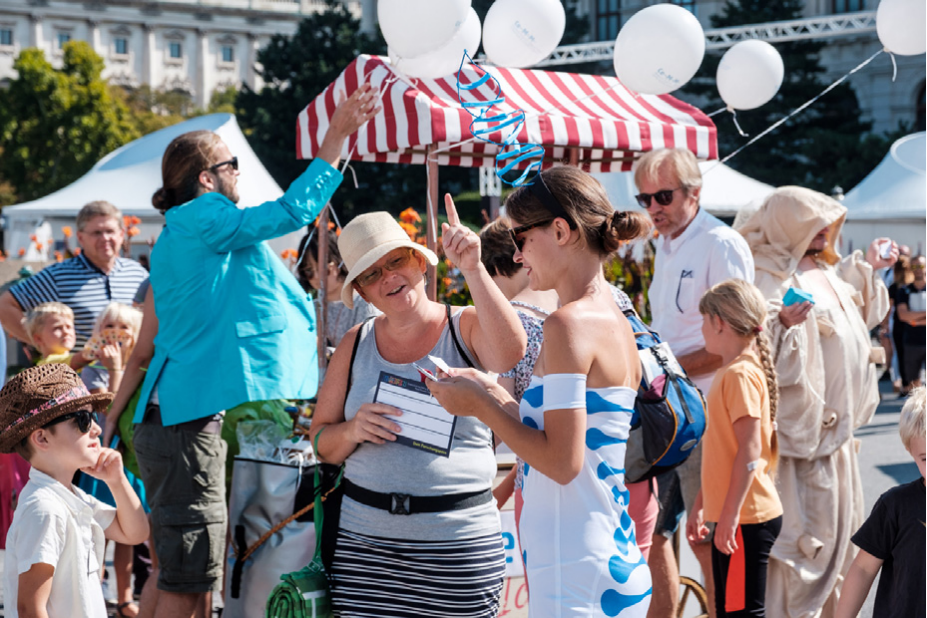
**“FWF BE OPEN” Science & Society Festival in Vienna, 2018. © Hans Leitner/CeMM**.

At the Research Center for Molecular Medicine (CeMM) of the Austrian Academy of Sciences, researchers and science communicators developed a Virtual Reality App specifically to promote the research at the institute. At its launch, CeMM created a printed quiz in the form of a card game that was distributed widely during the “BE OPEN—Science & Society Festival” in September 2018 (https://www.fwf.ac.at/en/about‐the‐fwf/be‐open‐science‐society‐festival). More than 30,000 citizens in total attended the festival (Fig 1). There CeMM team members invited visitors to download the app on their phone and play the quiz, using the cards as triggers, which generated good feedback. Since, the app has been further developed into an Augmented Reality version to teach younger audiences about the building blocks of life (DNA, proteins, cells) and provide users with insight into the human body as they zoom into the cell all the way down to the genome. Unfortunately, the number of people reached has not been as high as hoped for, due to a lack of funding and human resources to support the continued development. Nevertheless, the tool successfully served two purposes: informing others about the research being carried out at the institute and educating students about molecular biology.

Inspired by the popularity of reality TV, the Max‐Delbrück‐Center for Molecular Medicine (MDC) in Berlin, Germany, organized a live stream on YouTube from their laboratories as part of the 2020 Berlin Science Week. Each day from 9 am to 6 pm, scientists were filmed during their work with cameras rolling non‐stop, even though, at times, the lonely centrifuge whirring away was the only thing on display. The live TV format was interjected with special features, such as interviews with scientists and other staff, Q&A sessions with the public, plus “scientainment” events, such as Lab Olympics (“How fast can you pipet?”) and a music concert in the lab, for which lab equipment was used as music instruments. While around 150 people were watching at any given moment, the total reach was about 3,500 people, which is comparable to a regular open day at the MDC.

The Reality TV experiment was technically challenging and time and resource intensive. Yet, comments and questions received on YouTube indicated that it successfully conveyed the concentrated and yet fun atmosphere of a life science lab. Research work was demystified (“not much action”), biological concepts, and research explained, and career pathways to science were showcased. It also provided uncensored access to restricted areas and presented the scientists as they really are: a diverse, international group of people with ideas, dedication, and hobbies (https://www.youtube.com/watch?v=iNCZUEk3JGA&t=13s).

Each day from 9 am to 6 pm, scientists were filmed during their work with cameras rolling non‐stop, even though, at times, the lonely centrifuge whirring away was the only thing on display

Often it is challenging to reach audiences that are not interested in science. To address this, the Instituto Gulbenkian de Ciência (IGC) in Lisbon, Portugal, has been taking science to music festivals (Leão, & Castro, 2012). This initiative has been made possible due to a partnership between the IGC and “Everything is New”, the promoter of the NOS Alive Music Festival in Algés, near Lisbon, which is one of the top European festivals. IGC scientists and science communicators at the festival offer a broad range of activities, from speed‐dating with scientists to hands‐on activities, games, exhibitions, and demonstrations. A different theme is selected each year and specific activities designed accordingly. For example, in 2018, a darkroom was set up to create an environment where visitors could view biological samples under fluorescence microscopes and bioluminescent microorganisms. Over the three days of the festival, an average of 1,500 visitors interacted with IGC scientists, mostly teenagers, and young adults who can be otherwise difficult to reach through traditional school outreach activities.

## Consult

Consulting, the next level of stakeholder engagement, helps to inform research decisions, whether at a project or institutional level, and to strengthen relationships and trust.

In 2019, the Babraham Institute in Cambridge, UK, conducted a two‐stage public dialogue on genome editing in fundamental research. The first stage included development of the specification and methodology, followed by review and consultation with stakeholder representatives to set expectations, establish a common approach that would suit all stakeholders, and develop the materials for the second stage. The second stage involved a deliberative workshop in Cambridge over one and a half days. An agency specialized in market research and public consultation recruited the participants to ensure they were nationally representative. The recruitment of experts and scientists to participate in the workshops was challenging, as they had to donate a total of 12 h of their scarce free time without receiving any compensation.

The dialogue was organized as part of the European ORION Open Science project with the overall aim to explore public attitudes on genome editing, specifically within the context of fundamental research (https://www.orion‐openscience.eu/publications/reports‐papers/202103/public‐attitudes‐genome‐editing‐life‐sciences‐research). Another important objective was to understand how to better engage people from different backgrounds with potentially controversial scientific topics. All scientists who participated valued the opportunity to have in‐depth conversations with members of the public. One of them stated that “We need to acknowledge the knowledge of the public participants and recognise how it complements the expertise of the researchers to come to conclusions on how new technology can be introduced in a way that benefits society as a whole”.

If communication about science only pulls in those who are already interested, it fails to reach out to people who are most vulnerable to scientific misinformation. In order to address this problem, the IGC, the Instituto de Tecnologia Quimica e Biológica in Lisbon (ITQB NOVA), and the Oeiras municipality in Portugal organize a series of annual citizen deliberative forums. The focus of the first forum, held in February 2020, was how to make science accessible to citizens and involve citizens in science. Prior to the forum, an open “Idea Contest” was promoted through social media, posters, and fliers distributed through schools, senior universities, and local associations in Oeiras. Ten of the 30 submitted ideas were then selected by a jury. These ideas were later presented to the participants in the forum, which lasted two days and involved 30 citizens selected to reflect the demographics of Oeiras. During an initial learning phase, participants heard from three experts about a variety of approaches to science communication and public engagement and were also presented with the 10 winning ideas from the contest. With this knowledge and information, and based on their own personal experiences, participants discussed in small groups and designed their own proposals. In the final deliberation phase, the participants narrowed down the proposals to three that were then presented to a panel of decision‐makers, who gave feedback and discussed possibilities for implementation.

If communication about science only pulls in those who are already interested, it fails to reach out to people who are most vulnerable to scientific misinformation

Subsequent evaluation highlighted that the participants valued having their voices heard and were particularly interested in learning more about science and participating in the scientific process. The institutions promoting the forum found that the initiative gave them a greater insight into citizens' views, opinions, and wishes, and enabled them to gather unique innovative ideas to reach different audiences. However, promoting such forums can be very costly and time‐consuming for all parties and it is essential that the goals are clearly defined from the start to make sure that the citizen deliberative forum is the best tool. Finally, follow‐up of the proposals' implementation needs to be periodically communicated to participants and the general public to avoid creating disillusionment.

## Involve

The next level of engagement is involvement, whereby researchers collaborate with stakeholders, to listen to their concerns and aspirations and ensure these are addressed.

This was the rationale for an online public dialogue on research strategy organized by the Centre for Genomic Regulation (CRG) in Barcelona, Spain. The dialogue sought to gain opinions on fundamental research and discuss ethical and societal aspects with representatives from civil society and other stakeholders. The objective was to explore how to address societal views and concerns in the next CRG strategic plan (2021–2024). Thirty‐one citizens, who were selected by a market research company to reflect a representative sample of the Spanish population and who were financially compensated for participating, and 22 other stakeholders participated in a 13‐day online dialogue, owing to the COVID‐19 pandemic.

The exercise was highly valued by both the participants, who showed great interest in CRG research and operations and raised a number of relevant issues and expectations, and the scientists, who found the experience very enriching and altered their perception of how the public views them. Interestingly, some members of CRG's senior management initially expressed skepticism about involving citizens in the CRG's strategic plan. However, the organizers remained focused on the initial objectives of the dialogue and sought to respond to participants' opinions and requests, which thereby resulted in a high level of engagement. Importantly, the dialogue led to two key new actions being incorporated in the CRG's strategic plan: a series of regular talks on ethical implications of the latest technologies and two further public dialogues on future priority research topics.

Another example is the Embodying Memories project by the IGC (Matias *et al*, 2021). Using science and art, a multidisciplinary team of science communicators, researchers, and art education professionals designed a co‐creation project together with a community at risk of social exclusion. The main objectives were to increase awareness and engagement in science, and to improve the community's willingness to participate in new experiences, rather than aiming to increase the target audience's knowledge of a certain topic. The project was tailored to the needs and interests of the audience, a community of 14 women, aged between 64 and 84 with low literacy levels and socioeconomic status.

During the design phase, the communicators met with the participants and from these conversations the topic of neuroscience emerged. The implementation consisted of six indoor and two outdoor sessions. Each indoor session explored a different aspect of neuroscience, with different activities developed for each. The two outdoor sessions consisted of guided tours to an art museum (the Gulbenkian Museum in Lisbon) and a research center (the IGC). For the participants, this was their first experience of a museum and a research center, and the visits were greatly appreciated. The project results suggest that, in the short term, tailored science engagement programs can be effective in reaching and involving socially excluded publics. Yet, for medium‐term impact, such communities need to be further supported to facilitate access to cultural and scientific experiences.

The project results suggest that, in the short term, tailored science engagement programmes can be effective in reaching and involving socially excluded publics

Chronic liver disease is a major cause of morbidity and mortality worldwide; non‐alcoholic steatohepatitis (NASH) is an advanced type of non‐alcoholic fatty liver disease (NAFLD) which can lead to cirrhosis associated with liver fibrosis and progressive loss of function and increased risk of hepatocellular carcinoma. Early detection of NASH is key for prevention through lifestyle change and/or therapeutic intervention. The “Improving liver disease diagnosis” project, funded by the VIB Grand Challenges program in Ghent, Belgium, collected patient samples for analysis and storage to search for prognostic biomarkers for liver diseases. This project was inspired by the outcome of a previous initiative by the King Baudouin Foundation, in which several project partners were involved in a multi‐stakeholder dialogue initiative with physicians, citizens, patients, and biobank experts to set priorities within research on liver diseases and to discuss how biobanks could effectively help leverage this research (Raeymaekers, 2019). The results informed the VIB‐liver project in a number of ways to identify relevant research questions.

## Collaborate

**Figure 2:**
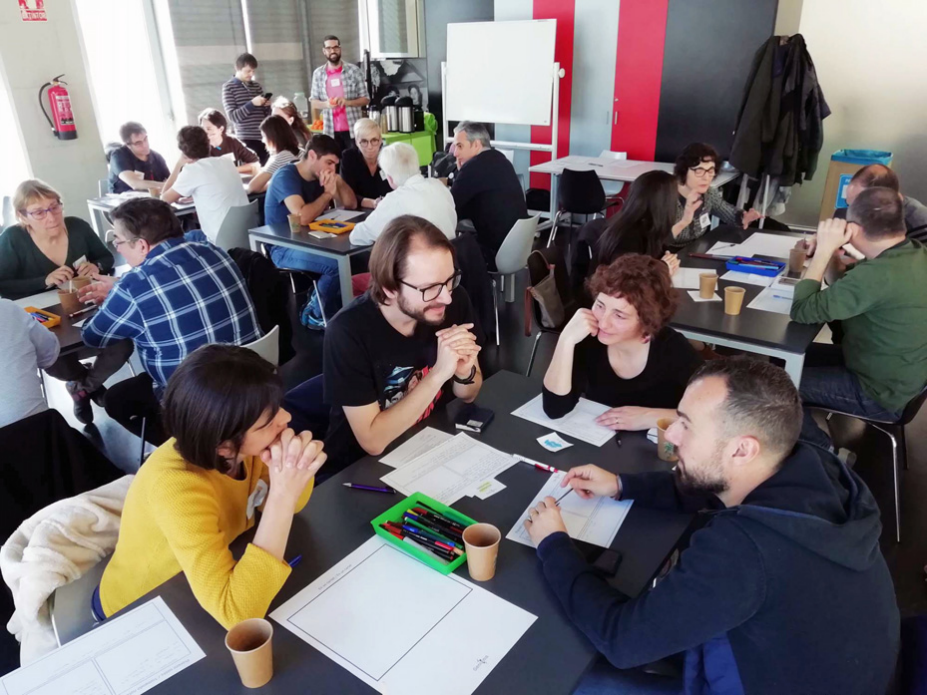
**Co‐creation event for the GENIGMA game with researchers, representative from patients' associations, storytellers, science communicators, and art creators. © Elisabetta Broglio/CRG**.

The GENIGMA citizen science project, developed as part of ORION Open Science, is an example of productive and intense collaboration between researchers and citizens from different interest and expert groups. GENIGMA is a game for smartphones to investigate 3D genomic structures and alterations in cancer cells. From the very start, the game itself was conceived with the help of teachers, representatives of patient associations, clinicians, designers, communicators, and gamers—more than 120 people participated in three different events (Fig 2). As the game developed, more citizens started to actively collaborate in the research project, initially by helping to test the validity of the data analysis pipeline and the game's functionality and later by analyzing the data, while they played. Despite initial skepticism and concerns about this highly collaborative approach, scientists subsequently recognized the value of including different perspectives. Participants' feedback indicated they were very happy to contribute their time and “non‐scientific” skills from the very beginning while their main motivation for participating was being able to help cancer research.

Soy is a subtropical crop ill adapted to Northern European climatic conditions. One major issue is the lack of (commercially available) nitrogen‐fixing bacteria in the soy plants' root nodules, which are necessary to achieve acceptable yields. Under the call “everything is everywhere”, the Belgian “Soy‐in‐1000‐gardens” project aimed to engage 1,000 citizens to grow soy in their gardens to “trap” nitrogen‐fixating bacteria in their root nodules for further analysis. Participants were recruited via “Mijn Tuinlab”, a platform for citizen science projects and via an official launch and its press coverage. More than 5,000 citizens applied and 1,154 were selected and provided with seeds. A digital interface collects data related to the growth of the soy plants. Additionally, the project also tested the effect of specific engagement actions (communication, information, etc.) on participants' overall awareness and attitude toward sustainable gardening, food consumption, and agriculture. Importantly, the project also included 100 farmers, who are introducing soy as a crop in Flanders. Communication with participants is key to the success of this kind of citizen science projects, and keeping the participants' community engaged is one of the main challenges.

## Empower

The holy grail of stakeholder engagement is to empower citizens to make fact‐based decisions. This was the ambition of the ORION Science and Art project ÆON “Trajectories of longevity and CRISPR”: to give citizens the impetus and the information to reflect on applications of the disruptive genome‐editing technology CRISPR/Cas9. Even though this was a fictional situation, it was an interesting exercise for critical thinking to empower the public to reflect on this specific question.

The holy grail of stakeholder engagement is to empower citizens to make fact‐based decisions

ÆON describes a futuristic scenario in which a CRISPR‐based rejuvenator exists. It gives the viewer a glimpse into the consequences of using vs. not using it by portraying a couple that made opposite decisions. The art piece shows what the couple looks like, how they interact with each other and with death, and forces the viewer to reflect on their own position. Through the additional display of the scientific proof‐of‐concept work, which shows that such a device, or at least a method for rejuvenation at the molecular level, could actually become reality, the viewer is also forced to think about the personal, social, and ethical implications.

ÆON was developed as part of an arts residency hosted by the MDC. Following an international Open Call process, the artist Emilia Tikka was selected to spend three months in the MDC labs, where, assisted by scientists, she carried out her experiments to produce a proof of concept for her futuristic scenario. The collaboration for ÆON was intense and a proof in itself that facilitation by a public engagement professional is key to success. In fact, this was the main challenge of the project: finding a common language and developing an understanding of each other's thinking as artists and scientists view the world differently and have different communication styles. Facilitation needs to be factored in for such projects to be successful: in the budget, in the project planning and the external communication activities.

**Figure 3:**
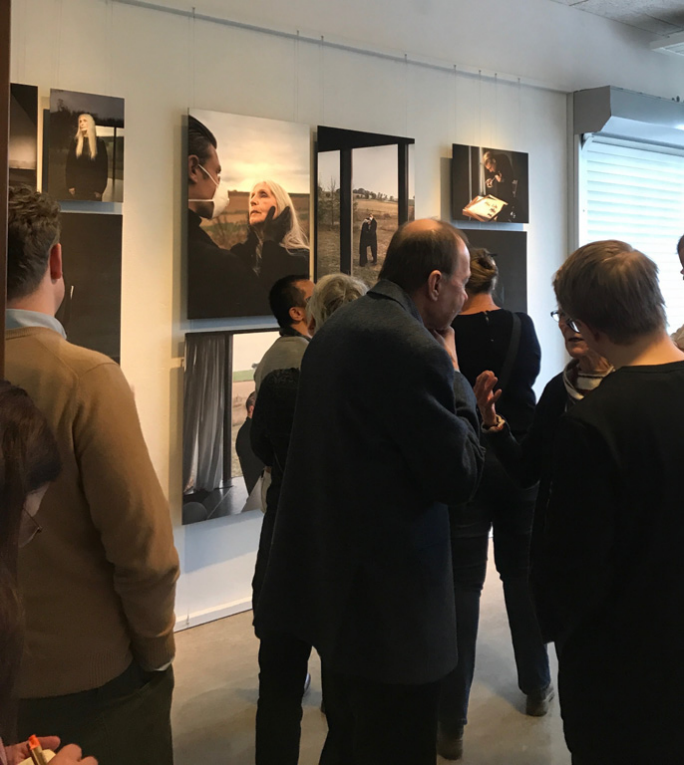
**Launch of the ÆON exhibition at the National Museum of Science and Technology in Stockholm, 2020. © Ben Libberton/Vetenskap & Allmänhet**.

ÆON has been on display in art galleries and exhibitions throughout Europe and received considerable press coverage. The art was also used as an impulse for public dialogues on genome editing in Sweden, Germany, Czechia, and the UK within the ORION Open Science project. The art piece was well received by the public and sparked lively discussions on what basic science can and cannot achieve in terms of societal decisions (Fig 3).

## Reflections

Based on our experience with public and stakeholder engagement in the life sciences, we have identified ten reflection points for researchers and institutions who are interested in engaging the public and other stakeholders in their research.

**Reflection 1**. A fundamental aspect is that stakeholder engagement activities must be framed within the strategy of the research institute or the leading organization. Senior management, public engagement experts, and scientists need to first reflect on what they want to achieve to define the objectives, the stakeholders with whom to interact, different engagement activities and necessary resources, such as personnel and funding. It helps to obtain commitment and buy‐in at all levels of the institution.
**Reflection 2**. The different levels of engagement should be considered as a continuum, and many projects may actually achieve different levels. Citizens are far better empowered to collaborate in the scientific process when they have been well informed, consulted, and involved. It is interesting to note that sometimes the level of engagement may increase during a project and even surpass the expected level. This was the case at the CRG, where the dialogues were initially planned as a consultation activity but developed toward the involvement level.
**Reflection 3**. It is important to reflect on the motivations of the target audience and the researchers who participate, and offer appropriate incentives to ensure their engagement. A stakeholder mapping exercise should identify the key stakeholder groups according to their goals, motivations, expertise, and interests; it is not sufficient to decide to involve “the public” or “citizens” as they are a very heterogeneous group. In addition, it is important to recognize that some stakeholders are harder to engage than others and to think creatively about how to reach them, for example, partnering with civil society organizations, running activities at venues and events such as a music festival, or engaging market research companies to recruit a representative group of participants.
**Reflection 4**. We cannot continue to pretend that “talk is cheap” and rely on minimum efforts and investments. Some activities can be costly and/or might require external expertise. Ideally, institutes will have funding to support stakeholder engagement; in other cases, funding can be sought through national, European, or international calls. Time and flexibility in research grants are also important factors, particularly when stakeholder insights can influence the direction of research projects (Lavery, 2018).
**Reflection 5**. It is important to be clear about the expected level of stakeholder engagement in order to manage expectations and ensure credibility. Moreover, it is likely that the number of stakeholders who can be engaged will decrease as the engagement deepens. This is the case, for example, with public dialogues that involve a smaller number of citizens to ensure meaningful conversations and exchange of ideas.
**Reflection 6**. Another critical aspect is transparent communication of the results back to the participants. Specifically in the case of public dialogues, citizen science projects, or activities where stakeholders' involvement and opinions can impact the course of the research project, the participants should be kept informed. Recognizing the value of participants' contributions and providing feedback on how their contributions will be used also increases the likelihood of a deeper level of engagement in the future.
**Reflection 7**. As unexpected behaviors or outcomes can occur during stakeholder engagement activities, it is therefore important to remain flexible and able to rapidly adapt to changing circumstances. A key part of engagement is “listening”, so attempts to control the direction that a discussion might take should be avoided at all costs. Instead, giving visibility to the existence of controversy but also highlighting emerging consensus in a safe, respectful, and friendly atmosphere will allow for more realistic and challenging views to emerge.
**Reflection 8**. Right from the start, it is important to discuss how engagement activities should be evaluated. Evaluation should focus on short‐ and long‐term impacts as well as what we can learn to inform and improve future work. This might require experts in social science, but if resources are limited, a simple evaluation will do too. Evaluation helps to collect evidence and to define best practices that others can learn from. There is always a risk that the desired impact is not achieved or that an activity may have negative consequences. Instead of being ignored or feared, such risks should be addressed through a risk assessment beforehand and mitigated by involving experts.
**Reflection 9**. Stakeholder engagement activity requires certain skills, and participating researchers should be provided with suitable training along with explanations of what is expected, what can happen and how it can be dealt with. If researchers fear being misinterpreted or that their message will be decontextualized for political/ideological reasons, specific support and training should be broadly provided by the institutions. This support can even evolve into a broader collaboration between researchers and professionals in science communication and engagement.
**Reflection 10**. Finally, as the principles of Open Science and Responsible Research and Innovation become embedded in the research process, stakeholder engagement should be included as part of academic productivity criteria to encourage researchers to dedicate the necessary time and resources to it.
